# Measuring disability in multiple sclerosis: the WHODAS 2.0

**DOI:** 10.1007/s11136-023-03470-6

**Published:** 2023-08-17

**Authors:** Carolyn A. Young, David J. Rog, Basil Sharrack, Cris Constantinescu, Seema Kalra, Tim Harrower, Dawn Langdon, Alan Tennant, Roger J. Mills, the Trajectories of Outcome in Neurological Conditions (MS) Study Group

**Affiliations:** 1 Walton Centre NHS Foundation Trust, Lower Lane, Fazakerley, Liverpool, L9 7LJ UK; 2 Institute of Systems, Molecular and Integrative Biology, University of Liverpool, Liverpool, UK; 3 Northern Care Alliance NHS Trust, Salford, UK; 4 Academic Department of Neurology, University of Sheffield, Sheffield, UK; 5 University of Nottingham, Nottingham, UK; 6 University Hospital of North Midlands NHS Trust, Stoke-on-Trent, UK; 7 University of Exeter Medical School, Exeter, UK; 8 Royal Holloway, University of London, Egham, Surrey UK; 9 Leeds Institute of Rheumatic and Musculoskeletal Medicine, University of Leeds, Leeds, UK

**Keywords:** Multiple sclerosis, Disability, Assessment, Rasch, Patient reported outcome measure, Trajectories of Outcome in Neurological Conditions (TONiC), World Health Organization Disability Assessment Schedule 2.0

## Abstract

**Introduction:**

Reliable measurement of disability in multiple sclerosis (MS) using a comprehensive, patient self-reported scale, such as the World Health Organization Disability Assessment Schedule (WHODAS) 2.0, would be of clinical and research benefit.

**Methods:**

In the Trajectories of Outcome in Neurological Conditions-MS study, WHODAS 2.0 (WHODAS-36 items for working, WHODAS-32 items if not working, WHODAS-12 items short-form) was examined using Rasch analysis in 5809 people with MS.

**Results:**

The 36- and 32-item parallel forms, and the cognitive and physical domains, showed reliability consistent with individual or group use. The 12-item short-form is valid for group use only. Interval level measurement for parametric statistics can be derived from all three scales which showed medium to strong effect sizes for discrimination across characteristics such as age, subtype, and disease duration. Smallest detectable difference for each scale was < 6 on the standardised metric of 0–100 so < 6% of the total range. There was no substantial differential item functioning (DIF) by age, gender, education, working full/part-time, or disease duration; the finding of no DIF for time or sample supports the use of WHODAS 2.0 for longitudinal studies, with the 36- and 32-item versions and the physical and cognitive domains valid for individual patient follow-up.

**Conclusions:**

Disability in MS can be comprehensively measured at interval level by the WHODAS 2.0, and validly monitored over time. Routine use of this self-reported measure in clinical and research practice would give valuable information on the trajectories of disability of individuals and groups.

**Supplementary Information:**

The online version contains supplementary material available at 10.1007/s11136-023-03470-6.

## Plain English summary

Patient care and research in multiple sclerosis (MS) would be greatly improved if it was possible to comprehensively measure disability by patient self-report. The World Health Organisation developed the self-reported Disability Assessment Schedule 2.0 (WHODAS), measuring cognition, mobility, self-care, getting along, life activities, and participation. In this study of 5809 people with MS taking part in the TONiC study, we showed that the WHODAS can be reliably used in MS, importantly, results can be easily converted to a standardised format which allows change to be measured. We checked two versions of the WHODAS, one for those who are not working (32 questions) and another for those in work/education (adds 4 questions). We proved these can be used interchangeably, so disability could be followed for individuals or groups over years as they move between education, work and not working. The short-form (12 questions) is accurate only for groups, not individuals. It would be simple to add regular assessment with the WHODAS to routine follow-up, providing valuable information to guide care.

## Introduction

The COVID19 pandemic has accelerated the move to increased remote assessment and monitoring of people with multiple sclerosis (pwMS). This has strengthened interest in identifying reliable and simple methods of measuring disability which do not require pwMS to attend in person, or clinicians to attempt disability assessment by phone or video.

Can measures traditionally assessed through clinical examination be adapted? The Expanded Disability Status Scale (EDSS) has been investigated for patient self-report in varied formats [1] but has psychometric weaknesses (see review [2]). Furthermore, a qualitative study with pwMS found that cognition and participation, topics not well measured by the EDSS, were important components of MS disability [3]. A more recent qualitative study of patients’ perceptions of outcome measures found that they should be comprehensive and measure neurological symptoms, cognitive impairments, mental health and well-being, self-care activities and social challenges, in order to adequately capture and support the needs of pwMS [4]. This requires either a range of patient-reported outcome measures (PROMs) or PROMs with sufficient breadth of coverage.

The World Health Organization (WHO) developed the WHO Disability Assessment Schedule WHODAS 2.0 as a comprehensive measure of disability, suitable for clinical practice or measurement at the population level [5]. It has been widely adopted; a systematic review on the use of the WHODAS 2.0 identified 810 studies [6]. All versions of the WHODAS 2.0 cover six domains: cognition, mobility, self-care, getting along, life activities, and participation. In the 36-item version (hereafter termed WHODAS-36), life activities encompass both household and work responsibilities. For use with people who are no longer working, four items related to work have been removed from this ‘life activities’ domain to create the 32-item WHODAS 2.0 (hereafter termed WHODAS-32). In order to create a more concise measure, two items for each of the six domains have been used to create the 12-item WHODAS 2.0 (hereafter termed WHODAS-12) [7].

There has been relatively little work exploring the structural validity of the WHODAS 2.0 in pwMS. One exploratory factor analysis of the WHODAS 2.0 in the Persian version for MS patients identified seven factors but the Confirmatory Factor Analysis did not comply with published requirements for acceptable fit [8, 9]. Another study looked at the earlier WHODAS II scale using Rasch analysis in 136 pwMS and found it a reliable and valid instrument for the assessment of patient-reported disability in MS [10].

The use of generic as opposed to disease-specific measures has been long discussed [11]. One advantage of generic measures such as the WHODAS 2.0 is that comparisons with non-MS populations are possible. The Nurses’ Health Study found that women with MS had a consistently lower physical ability than unaffected women [12]. Physical functioning gradually decreased with increasing age in both groups, but pwMS declined 3–4 times faster in midlife, while decline was similar in old age. This would indicate a relatively rapid decline in physical functioning during the key period when engaged with work. As an example, the physical function score of 45-year-old women with MS was comparable to that of 75-year-old unaffected women [12].

The current study examines the structure of the WHODAS 2.0 in its various formats in a calibration sample of 1050 MS patients recruited into the longitudinal Trajectories of Outcome in Neurological Conditions (TONiC) study, applying the Rasch measurement model [13]. It then describes the various WHODAS 2.0 scores across key demographic and clinical factors in the full sample of 5809 pwMS. Trajectories of disability in both an inception cohort and the full sample are explored. The aims are to investigate the capacity of the patient self-reported WHODAS 2.0 to measure the disability in pwMS at individual and group level, ideally enabling generation of interval level data which can be validly analysed by parametric statistics, with adequate effect sizes across both demographic and clinical groups.

## Methods

### Samples

Participants were recruited into the Trajectories of Outcome in Neurological Conditions-MS (TONiC-MS) study from hospitals and community teams in 33 collaborating sites across the UK (https://tonic.thewaltoncentre.nhs.uk/). Eligibility criteria included adults (aged over 18 years, and with no upper age limit) with physician-verified MS (by McDonald criteria [14]) of any disease subtype and level of disability, providing they could give informed consent and complete questionnaire packs (with the help of a scribe if necessary).

Data on disease subtype at time of study entry were provided by clinicians involved in the patients’ care and classified as relapsing–remitting (RRMS), primary progressive (PPMS), and secondary progressive (SPMS). Duration since diagnosis and Expanded Disability Status Scale (EDSS) band was recorded from the medical records. Informed consent was obtained from all participants prior to enrolment.

Participants completed a baseline questionnaire pack containing a number of PROMs (for list, see study protocol schematic in Supplementary File 1). Follow-up questionnaire packs were sent at intervals of at least 9 months. In total, the baseline and up to four follow-ups were included in the current data cut.

For analytical purposes, additional samples were drawn from the overall sample. The Rasch analysis used a calibration sample of 1050 cases, where 350 cases were randomly selected from each of first three time points in such a way that no individual appears in the total sample more than once [15]. The calibration sample was drawn from participants who had completed all 36 items of the WHODAS 2.0, so omitted those who had missing values on the four work items (except where they were imputed, see below). In addition, the data were further randomised into ‘training’ and ‘validation’ samples for cross-validation purposes. An inception cohort was also drawn from the full sample, being those pwMS with a duration 2 years or less years.

Ethical approval was granted from research committees (reference 11/NW/0743).

### Outcome measures

#### WHODAS 2.0

The scoring instructions for the WHODAS 2.0 define three specific scores: (1) items within a domain are summated to give the simple domain score; (2) domains are summated to give the simple total score; and (3) the total score is standardised to 0–100 [16]. The simple score range of the WHODAS-36 is 0–144, 0–128 for the WHODAS-32, and 0–48 for the WHODAS-12, where higher scores measure worse disability.

For the WHODAS-36, where respondents chose to skip the four items related to work and also reported that they were medically retired, then the item responses for the set were imputed to the response ‘extreme or cannot do’. Otherwise, items affected by the skip were flagged as missing. The same imputation was made to the one work item in the WHODAS-12. Only the 36 items were presented to the pwMS, and analysis then proceeded on the relevant set of items for each version.

#### EQ-5D-5L

The EQ-5D-5L utility value derived from 5 items scored 1–5; the range is from − 0.285 to 1, where higher scores indicate better health states [17, 18].

#### Neurological Fatigue Index-MS (NFI-MS)

The 10-item summary scale scored 0–30, where higher scores represent greater MS fatigue [19].

### Rasch analysis

Briefly, the data from each (sub)scale in each version of the WHODAS 2.0 were fit to the Rasch measurement model, to determine the internal construct validity, reliability, and invariance to key contextual factors [13]. These included age, gender, subtype of MS, duration, and time (baseline and first two follow-ups). Training and validation samples were included to further support invariance for the cross-validation analysis.

Full details of the Rasch methodology applied can be found in Supplementary File 2. Briefly, a hierarchical analysis strategy was employed starting with the summation of individual items (Level 1), through subscale scores or clusters of locally dependent items (Level 4), to parallel forms (Level 6) (Supplementary File 2: Table S1). The requirements of the Rasch model, elucidated in Supplementary File 2, must be met for any level of solution. A conceptual-based component approach can also be used where domains show local dependency, and these can be grouped into some overarching concept such as physical or social. Furthermore, at any level of analysis with local item dependency, a bi-factor approach can be utilised when dependency has been accommodated. This approach is based on the concept of essential unidimensionality and derives its estimate from what can be considered as the first common factor. The proportion of variance retained in that solution is reported.

### Trajectory analysis

To illustrate the use of the WHODAS 2.0 as a measure of overall disability, the total standardised score for the WHODAS-36 was examined over time of follow-up for the inception cohort and WHODAS-32 for the full sample, the latter adjusted for duration at baseline. Full details of the methodology are given in Supplementary File 2. Briefly, the time metric was the median month since the baseline questionnaire at each follow-up. Disability was assessed at baseline and up to four further follow-ups and modelled with a censored normal distribution.

### Additional attributes

The standard error of measurement (SEM) was calculated as SD*√(1 − reliability) and the smallest detectable difference (SDD) as ± 1.96*√2*SEM. The latter is the smallest statistically significant change in measurement results. The magnitude of effect sizes followed Cohen’s recommendations [20]. For the Rasch analysis, the effect size for substantial differential item functioning followed the simulation from Rouquette [21].

Participants were categorised as early onset if their date of diagnosis was before age 18 years and late onset if diagnosis was made after age 50 [22, 23]. The Index of Multiple Deprivation score gives an overall score for the relative level of multiple deprivation by area of residence of the participant [24].

Finally, in the first follow-up questionnaire, patients were asked to state whether or not their health had become worse, stayed the same, or improved. The same applied to their disability. Thus an anchor-based responsiveness was calculated, based upon the different metric estimate of those reporting worse or better. An effect size of that difference was given. Test–retest reliability was also calculated based upon those indicating no change in their disability state.

## Results

### The samples

The characteristics of the full and inception cohorts are summarised in Table 1. The mean EQ-5D-5L utility value of those in the inception cohort (duration since MS diagnosis 2 years or less) was significantly better than those in the full cohort (0.762, SD 0.217, compared to 0.680, SD 0.253; *t* = − 9.97 (*df* 5795); *p* ≤ 0.001). Over two-fifths (43.8%) of those with RRMS were on disease-modifying therapy (DMT).

**Table 1:** Demographic and clinical characteristics of the different samples

Sample/characteristics	Full	Inception
*N*	5809	813
Mean age (SD)	50.1 (12.0)	43.2 (12.1)
% Female	73.8	72.4
Mean duration since diagnosis in years (SD)	11.1 (9.8)	0.6 (0.5)
% Subtype		
Relapsing remitting	65.9	81.8
Secondary progressive	23.0	4.8
Primary progressive	11.2	13.4
% EDSS band		
0–4	51.0	74.5
4.5–6.5	37.5	22.6
7–7.5	6.7	2.5
8–9.5	4.7	0.4
Mean EQ-5D-5L utility value (SD)	0.680 (0.253)	0.762 (0.217)

The mean EQ-5D-5L utility value varied considerably by disease subtype, ranging from 0.763 (SD 0.206) in RRMS to 0.511 (SD 0.261) in SPMS. There was also a significant difference in EDSS level by disease subtype; for example, EDSS level 0–4 ranged from 8.8% in SPMS, to 71.4% in RRMS (*χ*^2^ 2.1e+03(9): *p* < 0.001).

For the calibration sample, baseline age, duration, and health utility value were not significantly different to the remainder of the full sample (*t*-test *p* > 0.05), neither were gender, type of onset nor EDSS levels (*χ*^2^ *p* > 0.05).

### The Rasch analysis of WHODAS-36, WHODAS-32, and WHODAS-12

The total scores of all three versions showed adequate fit to the Rasch model. Reliability (alpha) was consistent with individual clinical use except for the WHODAS-12, which only supported group use. A summary of the strategy of fit of the data in the calibration sample from each version of the WHODAS 2.0 and its various domains is given in Table 2. Briefly, a level 1 solution is at the item level, level 4 is two clusters of domains, or two clusters of LD items, level 5 is by taking alternative items, and level 6 requires item deletion (for full details, see Supplementary File 2.1). Data were also analysed after grouping domains into two components: ‘cognitive-social’ incorporating the three domains of cognition, getting along and participation, and ‘physical’, incorporating the remaining three domains of mobility, self-care, and life activities.

**Table 2:** Solution strategy of fit to the Rasch model of domains, components, and total scores of all versions of the WHODAS 2.0

	Simple item summation		Bi-factor equivalent solution	
	With reliability ≥ 0.85	With reliability < 0.85	With reliability ≥ 0.85	With reliability < 0.85
WHODAS-36				
Domains				
Cognition	Items			
Mobility	Items			
Self-care				Super Items
Getting along		Item deletion		
Life activities				Testlet
Participation			Testlet	
Components				
Cognitive-social				Testlet
Physical		Items		
Total			Testlet	
WHODAS-32				
Total			Testlet	
WHODAS-12				
Components				
Cognitive-social				Testlet
Physical	Items			
Total				Testlet

*WHODAS* World Health Organization Disability Assessment Schedule; *Items* Fit at the item level; *Testlet* Fit with testlets; *Super Items* Fit with alternative items; *Item deletion* remove one or more items

Applying the different levels of strategy for fit it was possible to fit the data to the Rasch model. The bi-factor equivalent solutions for the total scores of the WHODAS-36 and WHODAS-32 only discarded 8% of the variance to provide a unidimensional latent estimate, whereas the WHODAS-12 discarded more (average 13% across the two training and validation samples).

Detailed analyses for all results are provided in Supplementary File 3. Transformation tables for the different versions are available in Supplementary File 4, utilising the complex standardisation approach, giving each version a score of 0–100, to allow comparison across versions.

**Fig. 1:**
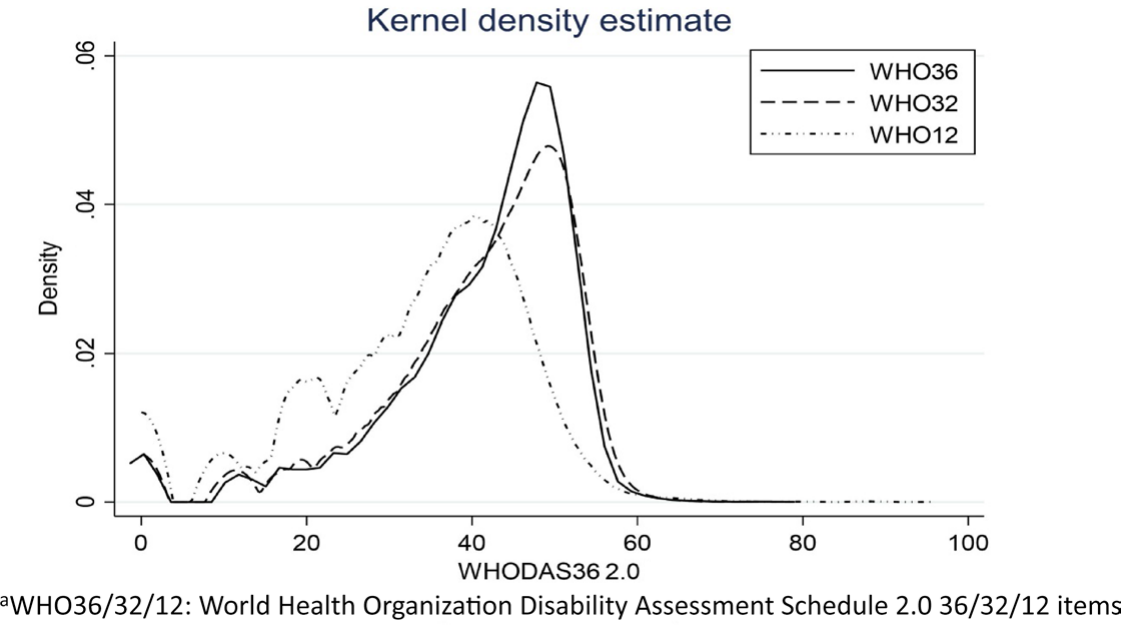
Comparative distributions of WHODAS 2.0 versions

Figure 1 shows the distribution of the three versions using the standardised score using a Kernel density estimate. While the WHODAS-36 and WHODAS-32 are equivalent, this is not the case for the WHODAS-12, which has a much lower mean score, and hence, its distribution is left-shifted from the other versions. Where the WHODAS-12 scores zero if the subject truly had no disability, they should also score 0 on the WHODAS-32; however, this was not the case and for those subjects with WHODAS-32 score > 10, items not in the WHODAS-12 such as ‘Remembering to do important tasks’ or ‘How much time did you spend on your health condition, or its consequences’ were gaining points on the WHODAS-32.

### Discrimination of WHODAS 2.0 versions across key demographic and clinical characteristics

With the exception of gender, all three parallel forms of the WHODAS 2.0 showed medium to strong effect sizes for discrimination across key characteristics (Table 3). The discrimination was particularly strong across the EDSS, where the WHODAS-32 was marginally more discriminating than other versions, albeit all versions had a ‘strong’ effect size. The difference of means at baseline between WHODAS-36 and WHODAS-32 gave an effect size of 0.04, indicating that they are equivalent.

**Table 3:** Descriptive analysis of the different versions of the WHODAS 2.0 (standardised metric). Full sample at baseline

Attribute	WHODAS-36	WHODAS-32	WHODAS-12	N
Age group (years)				
< 44 [A]	37.3	36.5	27.8	1634
44–51	40.6	40.0	32.3	1466
52–59	43.1	42.6	35.5	1404
60 + [D]	43.8	43.7	36.7	1299
Effect size A:D	0.56	0.69	0.66	
Gender				
Male	41.8	41.2	33.3	1521
Female	40.7	40.2	32.6	4281
Effect size	*0.10*	*0.14*	*0.06*	
MS subtype				
Primary progressive	44.5	44.6	37.6	649
Relapsing remitting	38.4	37.5	29.3	3823
Secondary progressive	46.7	47.1	40.6	1331
Effect size RR:SP	*0.85*	*0.94*	*0.96*	
EDSS				
0–4.0 [A]	35.8	34.6	25.8	2963
4.6–6.5	45.8	45.7	38.9	2175
7.0–7.5	48.9	49.7	43.5	390
8.0–9.5 [D]	49.2	50.5	44.0	275
Effect size A:D	*1.41*	*1.66*	*1.62*	
Duration (years)				
0–2 [A]	37.9	37.2	28.3	1201
3–8	39.4	38.6	30.7	1641
9–16	42.1	41.6	34.3	1543
17 + [D]	44.3	44.1	37.3	1418
Effect size A:D	*0.57*	*0.59*	*0.65*	
Total (mean)	41.0	40.5	32.8	5803

### Standard error of measurement (SEM) and the smallest detectable difference (SDD)

The standard error of measurement (SEM) and the smallest detectable difference (SDD) of the WHODAS-36 was 1.971 and 5.466, respectively, on the standardised metric. For the WHODAS-32, the SEM was 2.068 and SDD 5.733, and for the WHODAS-12, the SEM was 2.360 and SDD 6.543. As these are all on the standardised metric, then the percent of the full operational scale required to get above error is the same as the SDD. In contrast, the % SDD for the EQ-5D-5L was 15.533 based upon a reported 0.92 ICC reliability [25].

### Responsiveness to change across baseline and first follow-up questionnaire

Table 4 shows the responsiveness of the different versions of the scale, and the effect size of the magnitude of difference reported between those reporting worse and better. For health, 27.4% reported a worse state while 11.6% reported better. For disability, 40.9% reported a worse state, and 5.7% reported better. All versions of the scale across both anchors showed a large effect size.

**Table 4:** Effect size of metric differences between those reporting health or disability as worse or better

	Worse	Same	Better	Effect size
WHODAS-36				
Health	1.535	0.298	− 1.810	0.671
Disability	1.385	0.023	− 3.141	0.895
WHODAS-32				
Health	1.797	0.230	− 1.492	0.656
Disability	1.689	-0.074	− 3.144	1.030
WHODAS-12				
Health	2.332	0.990	− 1.482	0.531
Disability	2.053	0.756	− 2.759	0.645

Test retest reliability based upon those indicating no change in disability was 0.81, 0.82 and 0.83 for the WHODAS-36 -32 and -12, respectively.

### Inception cohort trajectory analysis using WHODAS-36

**Fig. 2:**
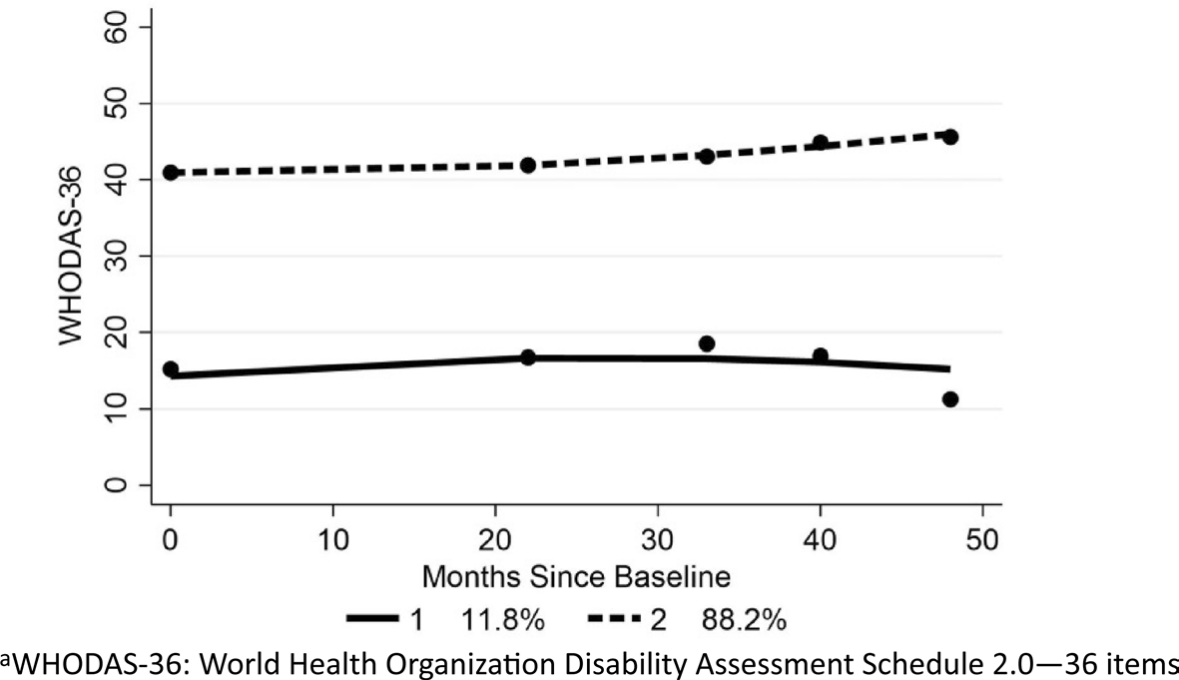
WHODAS-36 trajectories: inception cohort

Given the estimates derived from the Rasch analysis, an exploration was made as to how, if at all, the average levels of disability masked underlying groups with different trends over time. Two distinct WHODAS-36 trajectories were observed for those in the inception cohort (Fig. 2). The majority (88.2%) displayed a moderate level of disability with a gradual increase in the level of disability, but just over one in ten (11.8%) displayed a low level of disability, which remained low over time. Almost all of these low disability group (93.2%) were RRMS and were significantly younger than those with higher disability (39.0 vs 43.8 years) (*t* − 3.52 (*df* 811); *p* ≤ 0.001). There was no significant difference in gender, nor use of DMT between groups.

### Full sample trajectory analysis using WHODAS-32

**Fig. 3:**
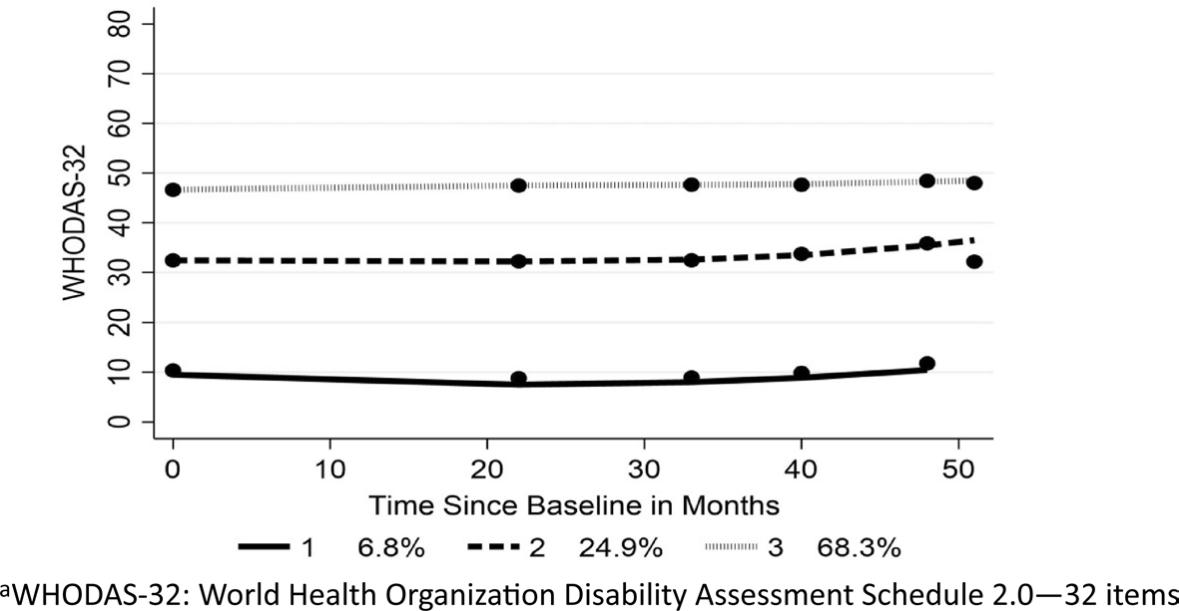
WHODAS-32 trajectories: full sample

Three trajectories were identified which satisfied the conditions given in Supplementary File 2 (Fig. 3). All three trajectories showed strong discrimination across all demographic, clinical, and other factors (Table 5). Of note, Groups 1 and 2, which showed quite different levels of disability over time, had equivalent durations. On the other hand, Group 3 members were much older, with a longer duration, and just 29.7% were in work, compared with 79.2% of those in Group 1.

**Table 5:** Baseline characteristics of three trajectory groups: full sample using WHODAS-32

	Trajectory Group			Significance		
	1	2	3	Total	*P*	*N*
WHODAS-32 (SD)	10.3 (8.9)	31.5 (6.0)	46.8 (5.4)	40.5 (11.9)	< 0.001	5799
Age in years (SD)	43.3 (11.2)	46.8 (11.8)	52.1 (11.6)	50.2 (12.0)	< 0.001	5809
Duration in years (SD)	7.6 (7.6)	7.5 (7.7)	12.7 (10.3)	11.1 (9.8)	< 0.001	5809
% Female	77.4	75.2	72.9	73.8	0.054	5808
MS subtype						
PP (%)	2.8	7.2	13.5	11.1		
RR (%)	94.7	87.0	55.3	65.9	< 0.001	5809
SP (%)	2.5	5.8	31.3	23.0		
EDSS (%)						
0–4.0	94.2	85.5	34.2	51.0		
4.5–6.5	5.1	13.8	49.4	37.5		
7.0–7.5	0.5	0.4	9.6	6.7	< 0.001	5809
8.0–9.5	0.2	0.3	6.8	4.7		
Health utility (mean)	0.949 (0.101)	0.871 (0.100)	0.583 (0.244)	0.680 (0.253)	< 0.001	5693
Fatigue NFI-MS (SD)	7.6 (5.6)	14.0 (5.1)	21.0 (5.2)	18.4 (6.7)	< 0.001	5545
In work (%)	79.2	66.5	29.7	42.2	< 0.001	5809
*N*	394	1446	3969	5809		

Subtype *PP* primary progressive; *RR* relapsing remitting; *SP* secondary progressive; *EDSS* Expanded Disability Status Scale; *NFI-MS* Neurological Fatigue Index-MS

All group comparisons are significant (*P* ≤ 0.001) with the exception of % female

A multinomial logistic regression explored which demographic, clinical, and other contextual factors may differentiate Groups 2 and 3 from Group 1 (Tables 6, 7). In summary, Groups 2 and 3 are older than those in Group 1, and more likely to be religious, more likely to have comorbidities, and be past and current smokers. Group 3 members are much less likely to be RRMS than Group 1 and to live in areas with higher deprivation. Of note, the relative risk of stage of onset (early or late) does not significantly vary between Groups 2 and 3, and Group 1. The Cragg–Uhler (Nagelkerke) pseudo-*R*^2^ was 0.27, indicating an adequate difference to the base model.

**Table 6:** Contrast between trajectory Group 2 and Group 1

Aspect	Relative risk	SE	*P*	Confidence Intervals	
				Lower	Upper
Group 2 relative to group 1					
Age	1.029	0.009	**0.002**	1.011	1.047
Male	1.089	0.179	0.605	0.789	1.502
Married	0.864	0.129	0.329	0.644	1.159
Religious/spiritual	1.416	0.225	**0.028**	1.038	1.933
Onset stage—early	2.070	1.238	0.224	0.641	6.683
Onset stage—late	1.009	0.296	0.974	0.572	1.781
Relapsing remitting	0.547	0.205	0.108	0.263	1.141
Secondary progressive	1.176	0.613	0.756	0.423	3.266
Duration	0.967	0.011	**0.003**	0.946	0.989
Comorbidity	1.715	0.238	** < 0.001**	1.308	2.251
Smoke—past	1.639	0.251	**0.002**	1.205	2.204
Smoke—current	1.930	0.512	**0.013**	1.147	1.781
Multiple deprivation	1.007	0.005	0.203	0.996	1.018

Multinomial logistic regression

Multiple Deprivation: Index of Multiple Deprivation 2011

**Table 7:** Contrast between trajectory group 3 and group 1

Aspect	Relative risk	SE	*P*	Confidence Intervals	
				Lower	Upper
Group 3 relative to Group 1					
Age	1.029	0.009	**0.001**	1.011	1.047
Male	1.167	0.186	0.334	0.853	1.596
Married	0.792	0.115	0.108	0.595	1.053
Religious/spiritual	1.680	0.259	**0.001**	1.242	2.274
Onset stage—early	0.989	0.600	0.985	0.301	3.248
Onset stage—late	0.742	0.211	0.297	0.425	1.298
Relapsing remitting	0.162	0.586	** < 0.001**	0.080	0.329
Secondary progressive	2.035	1.017	0.155	0.765	5.419
Duration	1.015	0.011	0.150	0.995	1.036
Comorbidity	3.041	0.412	** < 0.001**	2.332	3.966
Smoke—past	1.898	0.285	** < 0.001**	1.414	2.547
Smoke—current	3.689	0.942	** < 0.001**	2.237	6.084
Multiple deprivation	1.018	0.005	**0.001**	1.008	1.028

Multinomial Logistic Regression

Multiple Deprivation: Index of Multiple Deprivation 2011

## Discussion

The current study demonstrates that the total scores of the various versions of the WHODAS 2.0 can be used to measure disability in MS, through fit to the Rasch measurement model utilising a bi-factor equivalent approach. Thus, the WHODAS 2.0 provides a simple patient-reported outcome measure of disability, suitable for use by pwMS. Use of the transformation tables gives each version a score of 0–100, to allow comparison across versions; these interval level scores can validly be used for parametric analyses including change scores. The WHODAS-36 and WHODAS-32 can be seen as equivalent, with the latter used for people who are not working. If an individual being followed over the disease course moves between work/education and non-working status, their disability can be tracked seamlessly, using the WHODAS-36 during work/education and the WHODAS-32 when not working. Similarly, in a mixed sample of those in work or not, the appropriate transformation from the nomogram in Supplementary File 3 could be used for each version, obtaining the standardised score for all. This capacity to track disability over time, and across individuals and populations, allows analysis of changes linked to clinical or public health interventions. The total scores in both the WHODAS-36 and WHODAS-32 were robust enough for individual use. In contrast, the score on the WHODAS-12 cannot be considered equivalent to the larger versions; its reliability permits group use but is insufficient for individual use, and it may under-estimate some aspects of disability due to its abbreviated item set. All three versions of the scale showed good responsiveness to change.

The WHO conceptualises that every person is on a continuous spectrum of disability, or functioning, ranging from no disability/full functioning. Thus, WHO would not categorise individuals as disabled or not disabled, but place them on this continuous spectrum of functioning which can be measured using the WHODAS 2.0. The level of disability, or functioning, can be measured irrespective of the aetiology. For example, a person with MS and arthritis can have their mobility measured, and compared against those with MS alone or arthritis alone. The assessor is not asked to attempt to qualify how much mobility impairment is due to MS and how much to arthritis, or assume that comorbidities have no impact and disability relates to MS alone.

When disease-specific disability scales are employed, imprecision may occur when people are followed longitudinally if all disability is attributed to the disease and the effects of ageing and comorbidities disregarded. For example, if pwMS are followed over their life course, EDSS will be affected by natural changes of ageing, such as bilateral absent ankle reflexes in 8% of all adults aged 51–60 years and 30% of those aged 61–70, which would affect scoring of the EDSS pyramidal function [26]. When testing healthy subjects with no comorbidities or risk factors for sensory abnormality, 29% of over 65-years-olds have absent vibration sense at most distal joint compared to 1% of 18–64-year-olds [27]. This finding, occurring in over a quarter of healthy over 65 year olds, results in a score of 3 on EDSS sensory function. In a study measuring EDSS in 106 people, 55 years and older, with and without MS, median EDSS scores were 6.0 in people with MS and 3.0 in people without MS [28].

The WHO developed the WHODAS 2.0 to measure the person’s performance in a range of domains—cognition, mobility, self-care, getting along, life activities, and participation—which cover the breadth of human functioning. These domains of the WHODAS 2.0 mostly showed adequate fit although life activities had poor fit and reliability**.** However, the level of reliability of domains in the WHODAS-12 required a component approach to model fit, combining cognition, getting along and participation as one component termed ‘cognitive-social’, and mobility, self-care, and life activities as the other component termed ‘physical’. This offered some improvement in fit at the component level, and a better solution when the two components were used together to examine the total score. Thus, the WHODAS-12 is suitable for group use but not for tracking the disability, or functioning, of an individual over time.

The mobility and cognition domains in both the WHODAS-36 and WHODAS-32 were robust enough for individual use. Therefore, the cognition domain may have potential for simple, rapid monitoring of cognition-related disability in MS. A recent systematic review of the social consequences of MS disability found higher work disability in relation to higher physical disability and lower cognitive function [29]. Another study using latent class modelling of longitudinal cognitive data identified patients with worsening reaction times and increased risk of disability progression [30]. The authors argued that monitoring of cognition in clinical practice may enable detection of cognitive change trajectories and people with RRMS at risk of disability progression.

The WHODAS 2.0 showed good effect sizes across different ages, MS subtypes, EDSS band, and duration of disease. Trajectory analysis among 5809 people with MS showed three trajectories, which varied in their baseline characteristics of age, disease duration, MS subtype, EDSS band, health utility, fatigue, employment status, comorbidity, smoking history, religious or spiritual belief, and level of socio-economic deprivation. This would indicate that the 'average' trajectory of disability masks potentially important factors relevant to clinical management. For example, in the full sample while Groups 1 and 2 had similar durations, Group 1 showed a much lower level of disability, which was retained over four years. Does this indicate a possible ‘benign’ trajectory or different prodromal experience? In the inception cohort, a similar group with low disability was identified. What consideration should be given to the longer-term management of those who retain a low level of disability over time? A key implication of these findings is that any intervention based upon ‘average’ changes in disability may mask important differences in clinical path or influences of socio-economic factors, such as access to health care, which should be explored further [30].

Modelling using various registries has described mild, moderate, and severe disability trajectories in both PPMS and SPMS [31, 32]. A Swedish study examined trajectories of mean Sickness Absence and Disability Pension days per year (SA/DP) and found higher levels of SA/DP among pwMS of working age compared to the reference population [33]. Three trajectory groups of SA/DP were identified in pwMS: persistently low (55.2%), moderate increasing (31.9%), and high increasing (12.8%) [33].

There are a number of limitations to the study. As with all longitudinal studies, there is the problem of attrition, with the baseline and four further follow-ups requiring data collection 5 times over many years. The attrition appears worse for two reasons. The first is the average 22-month time between baseline and first follow-up. This was due to the administrative tasks of obtaining all the necessary ethical approvals to activate the follow-up stage and in the early part of the study resulted in a significant loss of participants. The second is that the current analysis is based upon a data cut in October 2019, and TONiC is an ongoing study. Therefore, what we have termed in a purist sense ‘attrition’ documents those individuals who had not provided follow-up data at that time point. However, the trajectory analysis accounted for differences in attrition across groups, and all other descriptive analyses were based upon the baseline data. The analysis of DIF also uses just single factors such as age or gender. No attempt was made to provide more complex factors such as age by gender.

The strengths of the study include the sample size; the calibration sample of 1050 people for the Rasch analysis, which delivered invariance for time and sample, together with interval scale data for analysis. The Rasch model itself is a strength, as it complies with fundamental measurement requirements, which include non-intersecting Item Characteristic Curves, and homogeneity, both of which are not available from other parametric IRT models. As a consequence, it provides traceability, a standard unit within its frame of reference, in this case pwMS, so allowing reliable and valid comparisons when measuring person ability [34]. Furthermore, the use of the WHODAS 2.0 itself, which has additional advantages in that it is built on the International Classification of Functioning, Disability, and Health (ICF) which is one of the recommended systems for e-health informatics [35–37]. Finally, this work provides transformation tables which allow users of the WHODAS 2.0 to transform their ordinal raw scores to interval level estimates based on the complex scoring metric of 0–100.

The study also raises issues for further research. It has been proposed to refine the concept of the MS continuum to include a prodromal phase which will help inform the true “at risk” period when considering exposures that might cause MS [38]. MS registries may have a part to play in this, although they can be limited in the range of information collected [39]. While different registries and studies may collect different information on health status, test equating may help to overcome the use of different PROMs, equating them onto a common reference metric [40]. In addition, the linking of the genome to the lived experience of MS explored through studies like TONiC may uncover genetic risk factors associated with the condition and the influence on how the disease manifests over the life course.

## Conclusions

These results show that the WHODAS 2.0 has considerable strengths for disability assessment and monitoring in pwMS. It can be collected by patient self-report and used at individual or group level to follow pwMS through life stages such as education, work or not working, without distortion from DIF and with a small SDD. Raw scores can rapidly be converted to interval level measurement, allowing use of parametric statistics such as means and change scores. It showed moderate to good effect sizes for key characteristics. The wide use of the WHODAS 2.0 in other diseases and healthy populations allows bench marking of disability and comparison to other populations. Incorporation of the WHODAS 2.0 into routine clinical follow-up and research would provide valuable information to guide care and contextualise research findings.

### Supplementary Information

Below is the link to the electronic supplementary material.

Supplementary file1 (PDF 681 KB)

## Declarations

### Data Access

Data supporting this study are not openly available due to reasons of sensitivity and are available from the corresponding author upon reasonable request. Data are located in controlled access data storage at Walton Centre NHS Trust. Please contact wcft.tonic@nhs.net.
