# Supplementary material for: Measuring disability in multiple sclerosis: the WHODAS 2.0
Source: Qual Life Res. 2023 Aug 17;32(11):3235–46. doi: 10.1007/s11136-023-03470-6 (PMC10522513; doi:10.1007/s11136-023-03470-6)
Supplement: Supplementary file 1 — Supplementary file1 (PDF 681 KB) [file 11136_2023_3470_MOESM1_ESM.pdf]

Measuring Disability in Multiple Sclerosis: The WHODAS 2.0
Supplementary Materials

Published in **Quality of Life Research 2023**

Carolyn A Young[1,2], David J Rog[3], Basil Sharrack[4], Cris Constantinescu[5], Seema Kalra[6], Tim Harrower[7], Dawn Langdon[8], Alan Tennant[9], Roger J Mills[1,2] on behalf of the Trajectories of Outcome in Neurological Conditions-MS Study Group

[1]Walton Centre NHS Foundation Trust, Liverpool, UK; [2]Institute of Systems, Molecular and Integrative Biology, University of Liverpool, Liverpool, UK; [3]Northern Care Alliance NHS Trust, Salford, UK; [4]Academic Department of Neurology, University of Sheffield, Sheffield, UK; [5]University of Nottingham, Nottingham, UK; [6]University Hospital of North Midlands NHS Trust, Stoke-on-Trent, UK; [7]University of Exeter Medical School, Exeter, UK; [8]Royal Holloway, University of London, Egham, Surrey, UK; [9]Leeds Institute of Rheumatic and Musculoskeletal Medicine, University of Leeds, UK.

Correspondence to: Prof CA Young. Email: Carolyn.young11@nhs.net. ORCID ID: https://orcid.org/0000-0003-1745-7720

Contents

Supplementary File 1 2

Schematic of Trajectories of Outcome in Neurological Conditions-MS (TONiC-MS) protocol 2

Supplementary File 2 3

2.1. Methods of Rasch Analysis 3

Supplementary File 2:Table S1. Strategies seeking fit of the data to the model. 5

2.2. Methods of Trajectory Analysis 5

2.3. References 6

Supplementary File 3 8

3.1. Detailed Results of Rasch Analysis 8

3.1.1. WHODAS-36 8

3.1.2. WHODAS-32 10

3.1.3. WHODAS-12 11

3.2. Comments on Granularity and Reliability 11

3.3. Cross Validation 12

Supplementary File 3: Table S1a. Fit of WHODAS-36, WHODAS-32 to the Rasch model in Training Sample. 13

Supplementary File 3: Table S1b. Fit of WHODAS-36, WHODAS-32 to the Rasch model in Validation Sample. 14

Supplementary File 3: Table S1c. Fit of WHODAS-12 to the Rasch model in Training and Validation samples. 15

Supplementary File 3: Table S2. Cross Validation in Calibration Sample, n=1050. 16

Supplementary File 4 17

Raw-Score-Interval Transformations 17

How to use this nomogram 18

Supplementary File 1

Schematic of Trajectories of Outcome in Neurological Conditions-MS (TONiC-MS) protocol

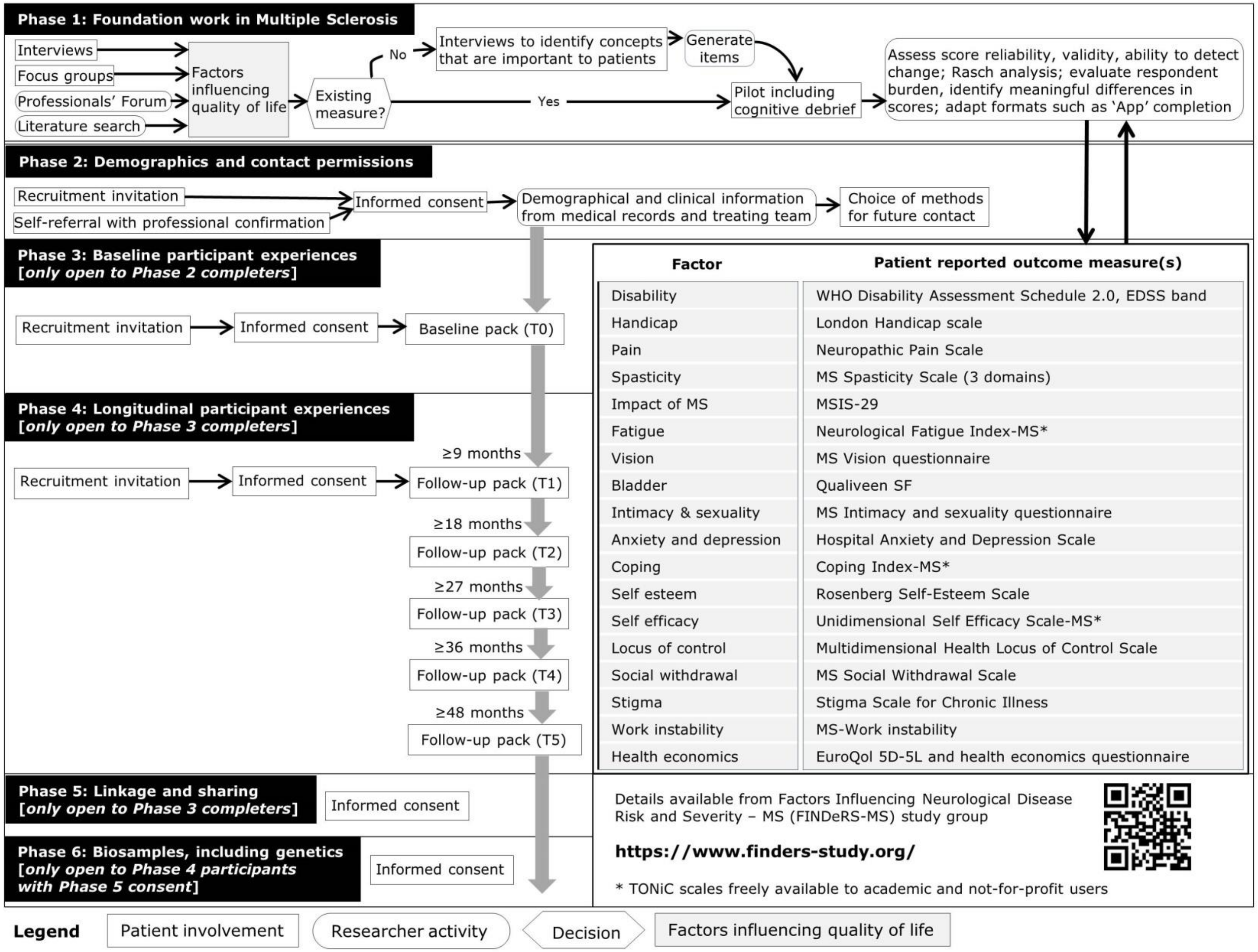

Supplementary File 2

2.1. Methods of Rasch Analysis

Data from each (sub)scale was tested against the requirements of the Rasch Measurement model [1]. Briefly, these requirements include: i) unidimensionality; ii) monotonicity; iii) homogeneity; iv) local independence; and v) group invariance [2, 3]. Whichever set of items are to be added together to provide a score, they should satisfy all of these requirements. They should: i) measure one thing (domain/ construct/trait); ii) the probability of a positive response to an item (or in the case of polytomous items, the transition from one response category to the next) should increase with underlying ability, as should the total score [4]; iii) the same hierarchical ordering of items should hold for each level (or grouping) of the score [5]; iv) items should be conditionally (on the score) independent of one another [6]; and v) the response to items across different groups such as age or gender should, conditioned on the total score, be the same – referred to as (the absence of) Differential Item Functioning (DIF) [3].

Each requirement is tested. A t-test is used to determine if two separate groups of items deliver significantly different estimates, following the procedure given by Smith [7]**.** The hierarchical ordering of items across the scale is determined through a Chi-Square test of fit based on grouped scores. Monotonicity is evaluated through inspection of the item-category ordering. Conditional item dependence is determined though the correlation of residuals, where pair-wise correlations should not exceed 0.2 above the average residual [8]**.** Should clusters of locally dependent items be found, consideration is given to grouping these into 'super items' or testlets (simply adding them together to make one larger item, the latter based on *a priori* defined groups) to absorb the local dependency [9]. In the RUMM2030 software, this gives a bi-factor equivalent solution retaining a specified proportion of the variance. This "Explained Common Variance (ECV)" is reported, whereby a value less than 0.7 is indicative of requiring a multidimensional model, a value above 0.9 a unidimensional model, and the grey area in between, undetermined, requiring further evidence [10]**.** Consequently, value of the ECV at 0.9 and above is considered acceptable in the current analysis. If two parallel forms are created from either a subscale structure, if present, or from the pattern of local dependency in the item set, this requires a latent

correlation ≥ 0.9. This is consistent with the reliability required for individual use [11]. Consequently, valid parallel forms would require both their latent correlation to be ≥0.9 and the ECV to be ≥0.9.

Group invariance (DIF) is tested through an ANOVA of residuals for age, gender, duration since diagnosis, education levels, and whether or not the patient is self-employed or employed, and working full-time or part-time. Should DIF be identified it is tested by a comparison of person estimates from split and unsplit solutions to see if it is 'substantive' [12]**.** Where the difference is significant (a paired t-test), the result is reported as an effect size where a value higher than 0.1 is considered to represent substantive DIF [13]**.** If this is present, then the scale works in different ways for the contextual factor under consideration, and results are reported separately. Finally reliability is reported as both a Person Separation Index (PSI), and as Cronbach's alpha. If data is normally distributed they are equivalent, but otherwise PSI tends to be lower the more data is skewed. Values are treated the same, and so values below 0.7 would be described as low, as they do not support group use.

A hierarchical approach to seeking fit of the data to the model for existing scales is adopted, with level 1 as the priority (Supplementary file 2: Table S1). All aspects listed above must be met for any level of solution. Should the original data fail to fit the model at any level (i.e. at a level 5 solution), item deletion will be considered (level 6). If this fails, then level 7 will be utilised to test if the scale satisfies ordinal scaling; if not level 8 indicates failure.

Supplementary File 2:Table S1. Strategies seeking fit of the data to the model.

Level	Nature	Adjustments	Reporting		
			Chi-Square	ECV ≥0.9	Latent Correlation ≥0.9
1	Item-based	None	Interaction	No	No
2	Item-Based	Clusters for Local Item Dependency	Interaction	Yes	No
3	Domain-based	On existing sub-scales >2	Interaction	Yes	No
4	Parallel Form	On existing sub-scales ≤2, or<br>2 local dependency patterns or conceptual groups	Conditional	Yes	Yes
5	Parallel Form	On alternative items	Conditional	Yes	Yes
6	Item Deletion	On all original items Repeat Levels 1-5	Interaction	No	No
7	Mokken Scaling	On items if Unidimensional. Loevinger's coefficient H ≥0.4-moderate	No	No	No
8	Fail	No valid ordinal scale	No	No	No

2.2. Methods of Trajectory Analysis

A group-based trajectory model was applied, which is designed to identify groups of individuals following similar developmental trajectories [14, 15]). It was implemented through traj.ado in STATA17 [16]. The number and shape (via polynomial functions) of trajectories were determined by analysing one to five group models without covariates. To accommodate attrition, a ‘dropout’ model was applied, specified in its basic form of constant dropout across assessment occasions [17]. The Bayesian Information Criterion (BIC) was used to determine the best-fitting model, also with consideration for a useful and parsimonious model. Average posterior probabilities above 0.7 were also deemed to indicate optimal fit [18]. Missing data were handled using a maximum likelihood approach based on a missing-at-random assumption.

The syntax for this approach is derived from STATA ‘add-on’ . To obtain this insert following into the SATA command line:

```
. net from http://www.andrew.cmu.edu/user/bjones/traj

. net install traj, replace
```

The actual code used for the WHODAS was as follows:

```
/* WHODAS trajectories*/
traj , var (WHODAS32_tra0 WHODAS32_tra1 WHODAS32_tra2 WHODAS32_tra3)
indep (t0 t1 t2 t3) model (cnorm) min (0) max (128) order (2 2 1) dropout (1 1 1)
trajplot, xtitle (Time) ytitle (WHODAS)
```

2.3. References

1. Rasch G. Probabilistic Models for Some Intelligence and Attainment Tests. Chicago: The University of Chicago Press; 1980.
2. Gustafsson J. Testing and obtaining fit of data to the Rasch model. Br J Math Stat Psychol.1980;33(2):205-33.
3. Teresi JA, Kleinman M, Ocepek-Welikson K. Modern psychometric methods for detection of differential item functioning: application to cognitive assessment measures. Stat Med. 2000;19(11-12):1651-83.
4. Kang HA, Su YH, Chang HH. A note on monotonicity of item response functions for ordered polytomous item response theory models. Br J Math Stat Psychol. 2018;71(3):523-35.
5. Rost J. An unconditional likelihood ratio for testing item homogeneity in the Rasch model. Educ Res Perspect. 1982;9(June):7-17.
6. Wilson M. Detecting and Interpreting Local Item Dependence Using a Family of Rasch Models. Appl Psychol Meas. 1988;12(4):353-64.
7. Smith EV. Detecting and evaluating the impact of multidimensionality using item fit statistics and principal component analysis of residuals. J Appl Meas. 2002;3:205-31.
8. Christensen KB, Makransky G, Horton M. Critical values for Yen's Q3: Identification of local dependence in the Rasch model using residual correlations. Appl Psychol Meas. 2017;41(3):178-94.

9. Wainer H, Kiely G. Item clusters and computer adaptive testing: A case for testlets. J Educ Meas. 1987;24(3):185-202.

10. Quinn H. Bifactor Models, Explained Common Variance (ECV), and the Usefulness of Scores from Unidimensional Item Response Theory Analyses [Masters Thesis]. North Carolina: University of North Carolina at Chapel Hill; 2014.

11. Bland JM, Altman DG. Statistics notes: Cronbach's alpha. B M J. 1997;314:572.

12. Hagquist C, Andrich D. Recent advances in analysis of differential item functioning in health research using the Rasch model. Health Qual Life Outcomes. 2017; 15(1):181.

13. Rouquette A, Hardouin JB, Vanhaesebrouck A, Sébille V, Coste J. Differential Item Functioning (DIF) in composite health measurement scale: Recommendations for characterizing DIF with meaningful consequences within the Rasch model framework. PLoS One. 2019;14(4):e0215073.

14. Jones BL, Nagin DS. A note on a Stata plugin for estimating group-based trajectory models. Sociol Methods Res. 2013;42(4):608-13.

15. Mori M, Krumholz HM, Allore HG. Using latent class analysis to identify hidden clinical phenotypes. JAMA. 2020;324(7):700-1.

16. StataCorp. Stata Statistical Software. Release 15 ed. College Station, TX: StataCorp LLC; 2017.

17. Haviland AM, Jones BL, Nagin DS. Group-based trajectory modeling extended to account for nonrandom participant attrition. Sociol Methods Res. 2011;40(2):367-90.

18. Nagin DS, Odgers CL. Group-based trajectory modeling in clinical research. Annu Rev Clin Psychol. 2010;6:109-38.

Supplementary File 3

3.1. Detailed Results of Rasch Analysis

3.1.1. WHODAS-36

A. Domains

A.1. Cognition

The data had adequate fit to the model (Table S1a). One item, ‘learning a new task’ showed differential item functioning (DIF) for age where younger people had less of a problem. The effect size of the difference between the unsplit and split person estimates was 0.018, and so the unsplit solution was retained. Validation required a level 4 solution with the first three items and last two clustering (average correlation -0.19) (Table S1b).

A.2. Mobility

The item ‘walking a long distance’ displayed DIF by subtype (Primary Progressive MS). The effect size of the difference between the unsplit and split estimates was just 0.018, and so no further action was taken. The item set just achieved unidimensionality. Validation required a level 2 solution (1 in training sample), as two items showed local item dependency (LD).

A.3. Self-Care

Local item dependency was observed between ‘washing self’ and ‘getting dressed’. Two super items from the four self-care items were created to adjust for this. DIF by subtype (Primary Progressive MS) was evident, but the effect size of the difference between the unsplit and split estimates was 0.08, and so no further action was taken. Note that the bi-factor equivalent solution led to 27% of the variance being discarded and, even then, the latent correlation between the two super items was just 0.47. The PSI reliability was very low, due to the substantive floor effect on this domain. On this occasion the validation required a level 2 solution (4 in training sample), with two items showing LD.

A.4. Getting along

The ‘sexual activities’ item displayed substantial misfit, and was removed. Following this, fit to the model was adequate. No DIF was observed. The same solution was required in the validation sample, but on this occasion two items displayed LD.

A.5. Life Activities

The eight items of the ‘life activities’ domain proved a significant challenge, driven by the fact that the household and work items formed two distinct and strong clusters of local item dependency. Using these clusters as testlets enabled a weak solution, but where 30% of the variance had to be discarded to obtain a unidimensional latent estimate.

The household and work items were then examined as separate domains, only the latter of which reached adequate fit. The new household domain had substantial DIF on age, with an effect size of 1.6 in the difference between the unsplit and split solutions. The split solution offered a weak solution. In the validation sample, the same problems were identified in the total score, requiring 34% of the variance to be discarded to obtain a weak solution. DIF was observed for age, but the curves were indistinguishable and so no further action was taken. The household subset could also not be resolved in the validation sample, including attempts to resolve by item splitting of disease subtype. The four items did form a valid ordinal scale (Loevinger's coefficient 0.92). The new work domain gave adequate fit.

In summary, the total score of Life Activities offers a weak solution as about one-third of the variance had to be discarded to achieve a unidimensional latent estimate. Following splitting the Life Activities domain into work and household item sets, the work items satisfied the Rasch model, but the household items did not, retaining an ordinal structure.

A.6. Participation

Two pairs of locally dependent items were identified and made into one testlet, with remaining items into a second testlet giving adequate fit. No DIF was observed. The same solution was found for the validation sample, giving adequate fit.

B. Components

B.1. Physical

The physical component had relatively poor fit to the model, and displayed DIF for subtype on the 'getting around' item. However, the effect size of the difference of person estimates between the split and unsplit solution was just 0.04, and so no further action was taken, and the unsplit solution retained. The validation showed good fit to the model. DIF was evident for gender but the effect size of the difference between the split and unsplit solution was 0.09, and so no further action was taken.

B.2. Cognitive/social

The cognitive/social component had good fit to the model, but displayed DIF for subtype on the 'participation' item. However, the effect size of the difference of person estimates between the split and unsplit solution was just 0.03, and so no further action was taken, and the unsplit solution retained. A similar result appeared in the validation sample, with subtype showing DIF but with an effect size of 0.09.

C. Total

The Total score of the WHODAS-36 showed good fit to the Rasch model, given a bi-factor equivalent solution. There was DIF by disease subtype, notably where PPMS varied from the curve in both testlets. However, the effect size of the difference in person estimates between the split and unsplit solutions was 0.08, and so no further action was taken. Given the direction of difference across the two testlets (one above, one below), it is almost certain that this DIF cancelled out at the test level. A similar result was found in the validation sample, with DIF on subtype, but with an effect size of just 0.02.

3.1.2. WHODAS-32

The total WHODAS-32 showed good fit to the model under a component strategy. However, DIF was evident for disease subtype with Primary Progressive MS showing a higher problem on the physical testlet and Secondary Progressive MS a lower level on the cognitive-social testlet. The effect size of the difference in person estimates between the split and unsplit solution was 0.008, indicating no significant bias, and so

no further action was taken. The same solution was found in the validation sample but with no DIF.

3.1.3. WHODAS-12

Reliability (alpha) of the individual domains ranged from 0.71 to 0.94, and so a component approach was adopted. The total score had good fit to the model using the component strategy in the training sample (Table S1c). There was DIF by subtype; Primary Progressive MS showed a higher (worse) score on the physical, but lower on the cognitive/social component. However, the effect size of the difference between the split and unsplit solutions was 0.08, and so no further action was taken. For the cognitive/social component, there was DIF by subtype, but the difference in curves was trivial and no further action was taken. The physical component had adequate fit to the model, good reliability and no DIF.

The results were mostly repeated in the validation sample, where for the total with a component approach, fit was adequate. DIF was evident for disease subtype on physical, but the curves were indistinguishable, and no action was taken. Likewise for the cognitive/social component DIF was evident by disease subtype, but with an effect size of just 0.013. The physical component had good fit.

3.2. Comments on Granularity and Reliability

As the granularity of the analysis increased, so did the disturbance of the model, mostly caused by variations of local item dependency across samples (for example, no Local Dependency (LD) in the training sample, but a single pair in the validation sample), or by DIF. At the domain level there was variation in the level of reliability, particularly where there were significant floor effects, leading to a divergence between the Person Separation Index (PSI) which is affected by a skewed distribution, and Cronbach’s alpha, which is not. Nevertheless, all domains retained reliability (alpha) consistent with at least group use. The cognition and mobility domains had high reliability in their original format.

When the training and validation samples were merged (n=1050), at the total score level, the disturbances seen at all levels above were absent. Importantly, there was never any DIF for time at any level of analysis, nor was there DIF by sample in the pooled data, supporting both use in longitudinal studies and the cross-validation across samples.

3.3. Cross Validation

The training and validation samples were merged, and the total scores of the three versions examined for the total combined sample, and for time (Table S2). All three versions showed good fit to the model, and cross validation was supported by the absence of DIF across samples. Furthermore, there was no DIF by time, supporting the use of the scale in longitudinal studies.

Supplementary File 3: Table S1a. Fit of WHODAS-36, WHODAS-32 to the Rasch model in Training Sample.

Scale Domains or Components	Residuals		Chi-Square		Reliability		Dime-nsion	DIF	ECV	Latent Corre-lation
	Item	Person	Value (df)	P	PSI	α	% t-tests (LCI)			
**WHODAS-36**										
**Cognition**	1.715	1.002	59.0(54)	0.297	0.84	0.91	3.1	Age	-	-
**Mobility**	1.513	0.852	49.3(45)	0.307	0.92	0.94	7.2 (4.9)	PP	-	-
**Self-Care**	0.227	0.775	15.6 (8)	0.049	0.50	0.88	0.4	PP	0.73	0.47
**Getting Along**	1.585	0.892	45.0(32)	0.064	0.59	0.88	1.5	None	-	-
**Life Activities**										
-Household	1.843	0.874	61.1(24)	0.000	0.94	0.97	2.9	Age	-	-
-Work	1.709	1.151	50.6(36)	0.054	0.83	0.99	3.2	None	-	-
-***Total***	1.085	0.672	37.7(24)	0.037	0.64	0.82	0.2	None	0.70	0.50
**Participation**	0.275	0.832	25.6(22)	0.270	0.79	0.89	1.8	None	0.92	0.83
**Components**										
**Physical**	1.702	0914	51.4(27)	0.003	0.85	0.83	4.0	RR	-	-
**Cognitive/ Social**	0.663	0.778	62.2(51)	0.136	0.58	0.79	0.8	RR	0.89	0.99
**Total**	0.276	0.876	106.0 (105)	0.453	0.81	0.93	1.9	PP	0.99	0.98
**WHODAS-32**	0.407	0.785	84.8(88)	0.577	0.78	0.88	3.7	PP/ SP	0.94	0.86
**Ideal Values**	**<1.4**	**<1.4**		**>0.01**	**>0.7**	**>0.7**	**<5%**		**>0.9**	**>0.85**

WHODAS: World Health Organization Disability Assessment Schedule 2.0; DIF: Differential Item Functioning; ECV: Explained common variance in testlet design; PSI: Person Separation Index; α: Cronbach's Alpha; LCI: Low confidence interval; PP: Primary Progressive MS; SP: Secondary Progressive MS; RR: Relapsing remitting MS

Supplementary File 3: Table S1b. Fit of WHODAS-36, WHODAS-32 to the Rasch model in Validation Sample.

Scale Domain or Components	Residuals		Chi-Square		Reliability		Dime-nsion % t-tests (LCI)	DIF	ECV	Latent Corre-lation
	**Item**	**Person**	**Value (df)**	**P**	**PSI**	**α**				
**WHODAS-36**										
**Cognition**	0.466	1.046	15.9(12)	0.195	0.87	0.90	1.96	None	0.94	0.86
**Mobility**	1.123	0.963	42.3(32)	0.105	0.90	0.91	5.81 (4.39)	PP	0.99	-
**Self-Care**	2.143	0.782	44.4(27)	0.019	0.58	0.79	0.2	None	0.80	-
**Getting Along**	0.328	0.998	32.5(26)	0.174	0.62	0.87	1.2	None	0.96	-
**Life Activities**										
-Household	1.643	0.875	38.5(20)	0.008	0.93	0.96	3.9	Age	-	-
-Work	2.414	1.061	57.6(36)	0.013	0.94	0.99	2.5	None	-	-
**-Total**	1.129	0.717	43.6(24)	0.008	0.60	0.79	0.2	Age	0.66	
**Participation**	0.412	0.855	32.1(22)	0.073	0.80	0.90	1.6	None	0.93	0.86
**Components**										
**Physical**	1.474	0.881	43.9(27)	0.021	0.85	0.82	3.6	Gender	-	-
**Cognitive/ Social**	0.385	0.872	54.8(54)	0.442	0.61	0.81	0.2	PP	-	-
**Total**	0.160	0.889	91.4 (104)	0.744	0.82	0.94	1.0	RR	0.99	0.99
**WHODAS-32**	0.598	0.894	114.4 (92)	0.057	0.86	0.98	0.0	None	0.91	0.84
**Ideal Values**	**<1.4**	**<1.4**		**>0.01**	**>0.7**	**>0.7**	**<5%**		**>0.9**	**>0.85**

WHODAS: World Health Organization Disability Assessment Schedule 2.0; DIF: Differential Item Functioning; ECV: Explained common variance in testlet design; PSI: Person Separation Index; α: Cronbach’s Alpha; LCI: Low confidence interval; PP: Primary Progressive MS; RR: Relapsing remitting MS

Supplementary File 3: Table S1c. Fit of WHODAS-12 to the Rasch model in Training and Validation samples.

Scale Sample	Residuals		Chi-Square		Reliability		Dimen-sion	DIF	ECV	Latent Correl-ation
	Item	Person	Value (df)	P	PSI	α	% t-tests (LCI)			
**Training Sample**										
**Components**										
**-Physical**	1.483	0.739	46.3(27)	0.012	0.83	0.88	2.5	None	-	-
**-Cognitive/ Social**	2.428	0.775	9.3(12)	0.677	0.35	0.69	0.6	RR	0.70	0.56
**Total**	1.582	0.733	31.4(34)	0.593	0.68	0.77	1.0	PP	0.85	0.70
**Validation Sample**										
**Components**										
**-Physical**	0.831	0.774	45.1 (27)	0.016	0.82	0.87	0.8	None	-	-
**-Cognitive/ Social**	2.302	0.731	21.6 (14)	0.086	0.47	0.74	0.8	PP	0.87	0.95
**Total**	1.664	0.794	47.9 (36)	0.023	0.71	0.77	0.2	Type	0.89	0.74
**Ideal Values**	**<1.4***	**<1.4**		**>0.01**	**>0.7**	**>0.7**	**<5%**		**>0.9**	**>0.85**

WHODAS: World Health Organization Disability Assessment Schedule 2.0; DIF: Differential Item Functioning; ECV: Explained common variance in testlet design; PSI: Person Separation Index; α: Cronbach’s Alpha; LCI: Low confidence interval; Type: undifferentiated disease subtype; PP: Primary Progressive MS; RR: Relapsing remitting MS * Inflated with unequal sized items sets

Supplementary File 3: Table S2. Cross Validation in Calibration Sample, n=1050.

Scale Sample	Residuals		Chi-Square		Reliability		Dime-nsion % t-tests (LCI)	DIF	ECV	Latent Correl-ation
	Item	Person	Value (df)	P	PSI	α				
**WHODAS-36**										
**Total**	1.336	0.791	108.4 (112)	0.579	0.75	0.85	4.6	None	0.92	0.82
**WHODAS-32**										
**Total**	0.338	0.776	106.3 (96)	0.221	0.76	0.88	2.2	None	0.92	0.81
**WHODAS-12**										
**Total**	2.258	0.771	43.1 (37)	0.225	0.69	0.77	0.6	None	0.86	0.71
**Ideal Values**	**<1.4***	**<1.4**		**>0.01**	**>0.7**	**>0.7**	**<5%**		**>0.9**	**>0.85**

WHODAS: World Health Organization Disability Assessment Schedule 2.0; DIF: Differential Item Functioning; ECV: Explained common variance in testlet design; PSI: Person Separation Index; α: Cronbach's Alpha; LCI: Low confidence interval

* Inflated with unequal sized item/testlet sets

Supplementary File 4

Raw-Score-Interval Transformations

Raw score	WHODAS-36	WHODAS-32	WHODAS-12
**0**	0.0	0.0	0.0
**1**	11.7	11.0	9.9
**2**	18.3	17.4	16.0
**3**	22.1	21.1	19.7
**4**	24.6	23.7	22.4
**5**	26.5	25.7	24.5
**6**	28.0	27.3	26.3
**7**	29.2	28.6	27.9
**8**	30.3	29.7	29.4
**9**	31.2	30.7	30.6
**10**	32.0	31.6	31.8
**11**	32.8	32.5	32.9
**12**	33.4	33.2	34.0
**13**	34.0	33.9	34.9
**14**	34.6	34.5	35.9
**15**	35.1	35.1	36.8
**16**	35.6	35.7	37.6
**17**	36.1	36.2	38.4
**18**	36.6	36.7	39.2
**19**	37.0	37.2	39.9
**20**	37.4	37.7	40.6
**21**	37.8	38.1	41.3
**22**	38.2	38.6	42.0
**23**	38.5	39.0	42.7
**24**	38.9	39.4	43.4
**25**	39.2	39.8	44.1
**26**	39.6	40.2	44.8
**27**	39.9	40.6	45.4
**28**	40.2	40.9	46.1
**29**	40.5	41.3	46.9
**30**	40.8	41.6	47.6
**31**	41.1	42.0	48.4
**32**	41.4	42.3	49.2
**33**	41.7	42.6	50.0
**34**	41.9	43.0	51.0
**35**	42.2	43.3	51.9
**36**	42.5	43.6	53.0
**37**	42.7	43.9	54.3
**38**	43.0	44.2	55.6
**39**	43.2	44.5	57.2

Raw	-36	-32	-12
**40**	43.5	44.8	59.0
**41**	43.7	45.1	61.1
**42**	43.9	45.4	63.6
**43**	44.2	45.6	66.6
**44**	44.4	45.9	70.1
**45**	44.6	46.2	74.4
**46**	44.8	46.5	79.9
**47**	45.0	46.7	88.0
**48**	45.2	47.0	100.0
**49**	45.5	47.2	
**50**	45.6	47.4	
**51**	45.9	47.7	
**52**	46.0	47.9	
**53**	46.2	48.1	
**54**	46.4	48.3	
**55**	46.6	48.6	
**56**	46.8	48.8	
**57**	47.0	49.0	
**58**	47.1	49.2	
**59**	47.3	49.4	
**60**	47.5	49.5	
**61**	47.7	49.8	
**62**	47.9	49.9	
**63**	48.0	50.1	
**64**	48.2	50.3	
**65**	48.3	50.5	
**66**	48.5	50.6	
**67**	48.7	50.9	
**68**	48.8	51.0	
**69**	49.0	51.2	
**70**	49.1	51.4	
**71**	49.3	51.5	
**72**	49.4	51.7	
**73**	49.6	51.9	
**74**	49.7	52.0	
**75**	49.9	52.2	
**76**	50.0	52.4	
**77**	50.2	52.5	
**78**	50.3	52.7	
**79**	50.4	52.9	
**80**	50.6	53.0	

Raw	-36	-32
**81**	50.7	53.2
**82**	50.9	53.4
**83**	51.0	53.6
**84**	51.2	53.7
**85**	51.3	53.9
**86**	51.5	54.1
**87**	51.6	54.3
**88**	51.8	54.5
**89**	51.9	54.7
**90**	52.1	54.9
**91**	52.2	55.0
**92**	52.4	55.3
**93**	52.5	55.5
**94**	52.7	55.7
**95**	52.8	55.9
**96**	53.0	56.1
**97**	53.2	56.4
**98**	53.3	56.6
**99**	53.5	56.9
**100**	53.7	57.2
**101**	53.8	57.5
**102**	54.0	57.8
**103**	54.2	58.0
**104**	54.4	58.4
**105**	54.6	58.7
**106**	54.8	59.1
**107**	55.0	59.5
**108**	55.2	59.9
**109**	55.4	60.3
**110**	55.7	60.8
**111**	55.9	61.3
**112**	56.2	61.9
**113**	56.4	62.4
**114**	56.8	63.0
**115**	57.0	63.7
**116**	57.4	64.4
**117**	57.7	65.2
**118**	58.0	66.0
**119**	58.4	66.9
**120**	58.8	67.9

Raw	-36	-32
121	59.3	69.0
122	59.7	70.3
123	60.2	71.8
124	60.7	73.5
125	61.3	75.9
126	61.9	79.1
127	62.6	85.9
128	63.3	100.0
129	64.0	
130	64.8	
131	65.6	
132	66.5	
133	67.5	
134	68.6	
135	69.7	
136	70.9	
137	72.3	
138	73.8	
139	75.5	
140	77.6	
141	80.2	
142	83.9	
143	89.9	
144	100.0	

How to use this nomogram

Providing the participant has answered all items in the scale, the scores assigned to each of the items - none (0), mild (1), moderate (2), severe (3) and extreme (4) – are added together; this summed total is called the raw score, and it is ordinal. To achieve an interval level estimate suitable for parametric analyses, read across the line to the appropriate column.

For example, if the WHODAS-36 was administered and all items answered, a total raw score of 115 is equal to an interval score of 57.0. If the WHODAS-32 was used, a total raw score of 115 is equal to an interval score of 63.7.
